# Assessing the Role of the Autonomic Nervous System as a Driver of Sleep Quality in Patients With Multiple Sclerosis: Observation Study

**DOI:** 10.2196/48148

**Published:** 2024-08-21

**Authors:** Max Moebus, Marc Hilty, Pietro Oldrati, Liliana Barrios, PHRT Author Consortium, Christian Holz

**Affiliations:** 1 Department of Computer Science Eidgenössische Technische Hochschule Zürich (ETH Zurich) Zurich Switzerland; 2 Neuroimmunology Department University Hospital Zürich Zurich Switzerland

**Keywords:** sleep quality, multiple sclerosis, autonomic nervous system, wearable sensors, mobile phone

## Abstract

**Background:**

Low sleep quality is a common symptom of multiple sclerosis (MS) and substantially decreases patients’ quality of life. The autonomic nervous system (ANS) is crucial to healthy sleep, and the transition from wake to sleep produces the largest shift in autonomic activity we experience every day. For patients with MS, the ANS is often impaired. The relationship between the ANS and perceived sleep quality in patients with MS remains elusive.

**Objective:**

In this study, we aim to quantify the impact of the ANS and MS on perceived sleep quality.

**Methods:**

We monitored 77 participants over 2 weeks using an arm-worn wearable sensor and a custom smartphone app. Besides recording daily perceived sleep quality, we continuously recorded participants’ heart rate (HR) and HR variability on a per-second basis, as well as stress, activity, and the weather (20,700 hours of sensor data).

**Results:**

During sleep, we found that reduced HR variability and increased motion led to lower perceived sleep quality in patients with MS (n=53) as well as the age- and gender-matched control group (n=24). An activated stress response (high sympathetic activity and low parasympathetic activity) while asleep resulted in lower perceived sleep quality. For patients with MS, an activated stress response while asleep reduced perceived sleep quality more heavily than in the control group. Similarly, the effect of increased stress levels throughout the day is particularly severe for patients with MS. For patients with MS, we found that stress correlated negatively with minimal observed HR while asleep and might even affect their daily routine. We found that patients with MS with more severe impairments generally recorded lower perceived sleep quality than patients with MS with less severe disease progression.

**Conclusions:**

For patients with MS, stress throughout the day and an activated stress response while asleep play a crucial role in determining sleep quality, whereas this is less important for healthy individuals. Besides ensuring an adequate sleep duration, patients with MS might thus work to reduce stressors, which seem to have a particularly negative effect on sleep quality. Generally, however, sleep quality decreases with MS disease progression.

## Introduction

Low sleep quality can lead to decreased mental and physical performance [1,2] and thus impacts quality of life. The autonomic nervous system (ANS) indirectly affects sleep because it regulates different physiological processes within the human body that affect the way we sleep, for example, respiration and heart rate (HR). The relation of the ANS to sleep quality is not well studied for diseases that are explicitly known to affect the ANS, such as multiple sclerosis (MS) [3], Parkinson disease [4,5], or Alzheimer disease [6,7].

For patients with MS, sleep quality is one of the main drivers for quality of life [8-11] besides disability, depressive mood, and fatigue. Sleep quality is often reduced in patients with MS due to cramps, pain, reduced mobility, spasticity, mucus retention, and restless leg syndrome [12]. Reduced sleep quality has been shown to increase the level of proinflammatory cytokines, which can result in a general worsening of symptoms (eg, fatigue or pain) [11]. Higher sleep quality has been linked to reductions in MS-related (secondary) fatigue [13,14].

Patients with MS are often affected by a dysfunction of the ANS [15]. Patients with MS have increased chances of observing symptoms of a dysfunctional ANS as early as 10 years before their diagnosis, indicating an early involvement of the ANS in disease progression [16].

HR variability (HRV) is considered a noninvasive measure for the activity of the ANS, for which it is one of the main biomarkers [17]. Owing to the evolution of mobile devices, such as smartwatches and fitness wristbands, their users can now reliably and continuously track HRV as well as other vital signs [18]. It is, therefore, possible to assess sleep quality using continuous data streams in real-life settings outside of sleep laboratories [19,20].

While laboratory-based polysomnography is still the gold standard of analyzing how humans behave while asleep [21,22], assessing sleep quality using continuous data streams in a real-life setting has many advantages. Using a vital tracker worn on the wrist, for instance, data can be recorded without great effort from the participants [21]. Studies can, thus, run for longer periods and unlock new data sources recorded outside sleep laboratories (eg, HR or step count during the day). The information acquired outside sleep laboratories using wearable sensors is more precise at a more granular level [23] than what can be assessed through questionnaires such as the Pittsburgh Sleep Quality Index [24].

In this study, we investigate how the activity of the ANS affects perceived sleep quality for patients with MS as well as a control group by constructing predictive models for subjective sleep quality based on continuous data streams collected over 2 weeks using a wearable sensor and a smartphone app. We thereby extend past studies that have investigated how HRV changes during sleep [18-20,25], how sleep quality (subjective and objective) is affected by factors such as stress [26-28], and how the ANS and HRV are affected by sleep disorders [29,30] and diseases such as MS [3,18,31]. In addition, we analyze the trade-off in performance between explainable and interpretable modeling techniques such as logistic regression and less easily interpretable techniques when modeling subjective response data. Techniques that are harder to interpret than logistic regression (eg, support vector machines, neural networks, or boosted decision trees) have been found to often outperform generalized linear models (GLMs) on medical data [32-34]. However, these methods are closer to so-called black box methods that are neither easily interpreted nor explained and have to be treated carefully when used for medical application [35].

## Methods

### Participants

For this study, patients diagnosed with MS aged between 18 and 65 years without concomitant diseases were recruited by convenience sampling at the neuroimmunology department outpatient clinic of the University Hospital Zurich, Switzerland. Patients with MS (n=53) and a control group (n=24) were recruited between November 29, 2019, and July 29, 2021. On average, participants were aged 35.8 (SD 10.1) years, and 48 participants were female. We ensured that for each patient with MS, there was at least 1 member of the control group with the same sex and within –5 to + 5 years of age. Overall, we tried to ensure a similar age distribution and sex ratio between the control group and patients with MS. As confirmed by Wilcoxon signed rank tests (Mann-Whitney *U* tests) post hoc, there is no significant difference in age or sex ratio between the 2 groups. With an average age of 36.8 years patients with MS are on average more than 3 years older than participants of the control group (33.5 years) corresponding to a *P* value of .11. In total, 35 out of 53 patients with MS are female compared to 13 out of 22 participants of the control group. The *P* value for differences in the sex ratio is 0.33.

In total, 2 patients with MS (not included in the count of 53) aborted the study. One patient aborted the study because of a medical emergency requiring stationary medical treatment. The other patient aborted the study because of feeling overwhelmed by the study procedure. Otherwise, all participants adhered to the study protocol.

We based our study size on the general recommendations for feasibility studies, which recommend numbers of 24 to 50 patients with MS [36-38]. We confirmed these estimates when determining the study size based on the precision of compliance rate estimates. Given the short duration of the study, we expected high compliance rates of >85%. We calculated that we would be able to estimate a participation rate as low as 85% to within –10% to +10% at a 95% CI based on a sample size as small as 49. Hence, we recruited via convenience sampling and aimed for at least 49 patients with MS. The high compliance rate was later confirmed with only 2 patients with MS aborting the study.

We included information about the medication of patients with MS in Multimedia Appendices 1 and 2. In particular, we listed medication that is known to affect HRV metrics and disease-modifying treatment (DMT).

### Ethical Considerations

The study protocol was reviewed and approved by the Cantonal Ethics Committee of Zurich (SNCTP000003494). All participants gave written informed consent, and procedures were in compliance with the Declaration of Helsinki.

### Data Set Description

For 2 weeks, the participants wore a wearable sensor on the arm (Everion, Biofourmis AG) to continuously record their HR, HRV, step count, and motion data from the arm. To ensure continuous data recording, participants were equipped with 2 Everion sensors. The Everion records HR and the total magnitude from a 3-axis accelerometer at 1 Hz as well as interbeat intervals (IBIs). We processed the raw IBIs identifying artifacts as proposed by Berntson et al [39]. We linearly interpolated all missing IBIs and chunked the continuous data stream into nonoverlapping 5-minute windows. We removed any 5-minute window with ≥5 interpolated IBIs. For all remaining 5-minute windows, we calculated the SD of the distance from the 45° line of the Poincaré plot of consecutive IBIs (SD1), the SD of the distance from the –45° line of the Poincaré plot of consecutive IBIs (SD2), and the SD of IBIs (SDNN).

Through a custom-developed smartphone app, participants logged their sleep quality each morning after waking up (Textbox 1) and stress levels continually during the day over the course of the 2 weeks. Participants logged their level of stress on a scale from 1 to 10. Participants received daily reminders to rate their quality of sleep and stress levels. We collected information about outside temperatures using an application programming interface service [40]. We equipped participants with a Google Pixel 3 (Google LLC) for the duration of the study in case their phone was not suitable to install our smartphone app (eg, they had an iPhone).

The continuous data streams (HR, HRV, motion, and step count) were aggregated per day depending on whether the participants were awake or asleep, extracting the average, minimum, and maximum (Table 1). In addition to these resulting nonstatic variables, which change on a day-to-day basis, we collected demographic information of each participant. For patients with MS, information about disease state, severity of MS-related disability, functionality of the ANS, and affection of the spinal cord was also collected. We modeled daily perceived sleep quality recorded each morning using nonstatic variables collected since the participants last woke up the previous day.

After the completion of the study, deidentifiable data were stored on secure, password-protected servers. The data are only shared with bona fide researchers upon reasonable request and after signing a data sharing agreement ensuring that the data privacy of all participants is protected and all data are stored securely.

**Explanation of self-reported sleep quality score.**

**Score and description**

- 5: Very refreshing
- 4: Rather refreshing
- 3: Moderately refreshing
- 2: Hardly refreshing
- 1: Not refreshing

**Table 1:** Collected data.

Source and name			Definition		Day or night
**Wearable sensor: Everion (Biofourmis AG)**					
	HR	Heart rate		Day and night	
	SDNN	SD of IBIs^a^		Day and night	
	SD1	SD of distance from 45° line of Poincaré plot of consecutive IBIs		Day and night	
	SD2	SD of distance from –45° line of Poincaré plot of consecutive IBIs		Day and night	
	Step count	Step count based on arm motion		Day and night	
	Sleep duration	Estimated based on acceleration and HR data		Night	
	Awake duration	Estimated based on acceleration and HR data		Night	
**Visual crossing [40] API^b^ service**					
	Temperature	Extracted via API using location recorded through smartphone app		Day	
**Prestudy questionnaires and medical assessments**					
	EDSS [41]	Expanded Disability Status Scale		—^c^	
	MSSS [42]	Multiple Sclerosis Severity Score		—	
	ARMSS [43]	Age Related Multiple Sclerosis Severity Score		—	
	ANS^d^ dysfunction	Assessed using COMPASS^e^ [44] questionnaire		—	
	MS^f^ diagnosis	Patient with MS or control group		—	
	MS type	None, progressive MS disease state, or relapse remitting MS disease state		—	
**Custom smartphone app**					
	Sleep quality	Scale from 1 to 5 as outlined in Textbox 1		Night	
	Stress	Self-reported multiple times a day a scale from 1 to 10		Day	
	Awake at night	—		Night	
	Sleep medication	—		Night	

^a^IBI: interbeat interval.

^b^API: application programming interface.

^c^Not applicable.

^d^ANS: autonomic nervous system.

^e^COMPASS: Computerized Pilot Aptitude Screening System.

^f^MS: multiple sclerosis.

### Data

A summary of all collected data can be found in Table 1.

We collected data mainly from 3 different sources: a wearable sensor, a custom smartphone app, and prestudy questionnaires or medical assessments. Nonstatic data were aggregated every day separately for when the participants were awake and asleep. For nonstatic variables, we calculated the minimum, mean, maximum, ratio of minimum to mean, and ratio of maximum to mean. For the static variables Expanded Disability Status Scale (EDSS), MS Severity Score (MSSS), Age-Related Multiple Sclerosis Severity Score (ARMSS), and ANS dysfunction, we used 3, 3, 4, and 17 as cutoff points, respectively, to transform them into binary variables. For the control group, we set these values to 0 before applying the cutoff rule. The custom smartphone app was distributed through the Google Play Store in Switzerland. Participants who did not own an Android phone were equipped with the Google Pixel 3 for the duration of the study.

### Data Processing

For the analysis of HR and HRV, we only included periods where participants were resting as recommended for photoplethysmography-based HRV measurements [45]. Participants were classified as resting during 5-minute windows if their HR (measured in bpm) was <0.55 × (220 bpm – age) [46].

Furthermore, we transformed HRV recordings to normative values considering age, sex, and time of day [47]. The recorded data per participant was split into daily intervals based on when the participants woke up and aggregated as outlined in Table 1.

The times when participants went to bed and woke up were estimated manually based on HR, acceleration data from the wearable sensor, and step count. To remove periods where the participants were in a transitional state between awake and asleep, we excluded 1 hour of data before and after the estimated going-to-bed and wake-up times.

All analysis was done in Python (version 3.8, Python Software Foundation). For modeling, we made use of the scikit-learn, keras, and XGBoost libraries [48-50].

## Results

### Overview

In this study, we analyzed the drivers of perceived sleep quality via predictive modeling. We first looked at significant differences in average perceived sleep quality between different subgroups of the participants. Subsequently, we compared the performance of different models for perceived sleep quality normalized per participant. Finally, we analyzed the variables where relative changes are calculated to significantly affect participants’ sleep quality compared to their personal average over the 2 weeks.

For the analysis, data were available on average for 19.7 hours per day per participant (ie, approximately 82% of the time, SD 2.64 hours). This was consistent across patients with MS and healthy controls. Besides nonwear, reasons for data not being available at all times include participants switching between the 2 devices they had been equipped with for charging and subsequent synchronization issues.

### Differences in Sleep Quality Across Subgroups

The average self-reported sleep score was 3.72 (scale from 1 to 5; Textbox 1). The distribution of responses is shown in Multimedia Appendix 3. Table 2 lists the observed mean differences and significances according to Wilcoxon rank sum tests for subgroups defined by sex, patient status, type of MS, dysfunction of the ANS, affection of the spinal cord, and severity of MS in terms of scores on the EDSS [41] and variations thereof (MSSS [42] and ARMSS [43]) measuring MS-related disability. We chose Wilcoxon signed rank tests (aka Mann-Whitney *U* test) over alternative methods, such as *t* tests, because they are rank based and distribution-free. Therefore, they are a natural choice for ordinal data (such as items on a Likert scale) as well as nonnormally distributed or binary data.

Table 2 shows the outcome of distribution-free 2-sided Wilcoxon signed rank tests for mean shifts in self-reported sleep quality between subgroups defined based on demographic information, patient status, and disease state. A higher reported sleep quality score corresponds to higher perceived sleep quality (Textbox 1). *P* values are calculated based on distribution-free 2-sided Wilcoxon signed rank tests for mean shifts in self-reported sleep quality between two groups of participants.

The perceived sleep quality score of female participants was significantly lower, indicating that their perceived sleep quality was higher than that for male participants. Participants with different types of MS did not report sleeping significantly differently. However, participants whose spinal cord was affected by lesions or with ANS dysfunction reported significantly lower perceived sleep quality—similar to participants scoring high on the MSSS, ARMSS, and EDSS scales. Apart from ANS dysfunction, the differences were not (as) significant when including the control group. However, for patients with MS, the severity of MS led to significant differences in perceived sleep quality.

**Table 2:** Mean comparison of the self-reported sleep quality score between different subgroups.

Group 1				Group 2			*P* value
Description, n (%)		Sleep quality score, mean (SD)	Description, n (%)		Sleep quality score, mean (SD)		
**Sex**							
	Female, 48 (62)	3.84 (0.60)	Male, 29 (38)		3.54 (0.62)	.01	
**Disease status**							
	No MS^a^, 24 (31)	3.71 (0.48)	Patients with MS, 53 (69)		3.73 (0.68)	.56	
	No MS, 24 (31)	3.71 (0.48)	MS type PMS^b^, 9 (12)		3.64 (0.57)	.73	
	No MS, 24 (31)	3.71 (0.48)	MS type RRMS^c^, 44 (57)		3.75 (0.70)	.43	
	MS type PMS, 9 (12)	3.64 (0.57)	MS type RRMS^c^, 44 (57)		3.75 (0.70)	.64	
**ANS^d^ dysfunction**							
	No dysfunction of ANS: all^e^, 53 (69)	3.86 (0.52)	Dysfunction of ANS^f^, 24 (31)		3.42 (0.73)	<.001	
	No dysfunction of ANS: MS^e^, 29 (38)	3.99 (0.52)	Dysfunction of ANS^f^, 24 (31)		3.42 (0.73)	<.001	
**Spinal cord status**							
	Spinal cord unaffected: all^e^, 53 (69)	3.82 (0.53)	Spinal cord affected^f^, 24 (31)		3.51 (0.77)	.10	
	Spinal cord unaffected: MS^e^, 29 (38)	3.91 (0.55)	Spinal cord affected^f^, 24 (31)		3.51 (0.77)	.06	
**MS-related disability**							
	MSSS^g^<3: all^e^, 54 (70)	3.80 (0.58)	MSSS≥3^f^, 23 (30)		3.55 (0.69)	.14	
	MSSS^g^<3: MS^e^, 30 (39)	3.87 (0.64)	MSSS≥3^f^, 23 (30)		3.55 (0.69)	.07	
	ARMSS^h^<5: all^e^, 42 (55)	3.84 (0.53)	ARMSS≥6^f^, 6 (8)		3.59 (0.70)	.17	
	ARMSS^h^<5: MS^e^, 18 (23)	4.00 (0.55)	ARMSS≥6^f^, 6 (8)		3.59 (0.70)	.05	
	EDSS^i^<3: all^e^, 59 (77)	3.77 (0.56)	EDSS≥3^f^, 18 (23)		3.58 (0.79)	.09	
	EDSS^i^<3: MS^e^, 35 (45)	3.81 (0.61)	EDSS≥3^f^, 18 (23)		3.58 (0.79)	.04	

^a^MS: multiple sclerosis.

^b^MS type PMS: progressive MS disease state.

^c^MS type RRMS: relapse remitting MS disease state.

^d^ANS: autonomic nervous system.

^e^A group called “condition: all” refers to all participants for whom the condition is true (patients with MS as well as control group). A group called “condition: MS” refers only to participants diagnosed with MS for whom the condition is true (ie, no control group).

^f^All participants for whom this condition is true were diagnosed with MS.

^g^MSSS: Multiple Sclerosis Severity Score [42]. Per definition of the MSSS, the chosen cutoff point distinguishes between light to no disability (≤3) and more severe implications (>3).

^h^ARMSS: age-related multiple sclerosis severity score [43]. Per definition of the ARMSS, the chosen cutoff point distinguishes between light to no disability (≤4) and more severe implications (>4).

^i^EDSS: Extensive Disability Status Scale [41]. Per definition of the EDSS, the chosen cutoff point distinguishes between light to no disability (≤3) and more severe implications (>3).

### Comparison of Different Modeling Techniques for Normalized Perceived Sleep Quality

Higher-dimensional “black box” methods have outperformed clearly explainable and interpretable techniques such as GLMs and thus have gained more and more popularity for medical applications. For binary classification, neural networks [33] and tree ensemble methods [51] tend to outperform logistic regression in recent literature. However, logistic regression naturally models the changes in odds for a binary outcome, allowing for very easy and clear interpretation. The following is a comparison of these modeling techniques as well as a generalized additive model (GAM) and support vector machine applied to model perceived sleep quality as part of our study.

To analyze how relative changes in input features affect perceived sleep quality compared to participants’ average responses, we normalized input features and the perceived sleep quality response. We subtracted the mean value per participant across the 2 weeks and divided by the respective SD for each participant. As the normalized sleep quality response failed the Shapiro-Wilk normality test (*P*<.001), thus violating one of the assumptions of linear regression, we transformed the problem into the binary setting. We split the data into high-quality and low-quality sleep based on whether a participant slept better than their personal average recorded during the study. The performances of the different models listed in Table 3 are similar (63%-67% accuracy). For all models, we used 0.5 as a cutoff point to classify between high- and low-quality sleep. The best performing model is the GAM, achieving an accuracy of 67% with an area under the curve (AUC) of 0.71. The support vector machine, logistic regression (GLM), and symmetric boosted trees achieve the same accuracy (65%). Of the 3, the support vector machine achieves the highest AUC with 0.70. Adding interaction terms to the logistic regression models (GLM of order 2 and 3) does not improve accuracy but only decreases AUC (from 0.69 to 0.66). In terms of accuracy, the neural network performs the worst out of the selected models with 63%. In terms of AUC, however, neural networks outperform boosted tree ensemble methods and GLMs with interaction terms.

While boosted trees and neural networks perform feature selection themselves, we constructed a sequential feature selection procedure for GLMs, the GAM, and the support vector machine.

**Table 3:** Model performances for predicting normalized perceived sleep quality using all available information recorded while participants were awake and asleep.

Model^a^ comparison	Accuracy^b^ (%)	Precision^b^ (%)	Recall^b^ (%)	AUC^c^
Support vector machine	65	66	64	0.70
Symmetrical boosted trees^d^	65	67	65	0.66
Neural network^e^	63	63	63	0.68
GLM^f^	65	66	65	0.69
GLM order 2^g^	65	65	65	0.68
GLM order 3^h^	65	65	65	0.66
Generalized additive model	67	67	67	0.71

^a^The models were evaluated on 50 perfectly balanced test sets, each consisting of randomly selected 20% of participants who were removed from the training set.

^b^Using a cutoff point of 0.5 for the calculated probabilities.

^c^AUC: area under the curve.

^d^Architecture chosen based on Bayesian optimization [52]: depth of 5 and 600 boosting rounds.

^e^Architecture chosen based on Bayesian optimization [52]: 2 hidden layers containing 16 neurons with hyperbolic tangent activation functions and dropout rates of 0.7 and 0.5, respectively.

^f^GLM: generalized linear model.

^g^In addition to the untransformed features, this model includes interactions between 2 variables.

^h^In addition to the untransformed features, this model includes interactions between up to 3 variables.

### Modeling Normalized Perceived Sleep Quality

In this subsection, we analyze logistic regression models for normalized perceived sleep quality without interaction terms. Although GAMs outperformed GLMs, they fit effects as smoothing splines, making model comparison harder than in the generalized linear setting where effects on the modeled OR are assumed to be linear. Per participant, perceived sleep quality and input features were normalized by subtracting the average per participant and dividing by the respective SD per participant. We constructed 3 models with different input features to compare the consistency of effects depending on what information is available to the model. The first model (M1.1: night and day) uses all available data recorded during the night and the previous day to model normalized perceived sleep quality. The second model only uses data recorded, while the participants were asleep (M1.2: night), such as HR while asleep. The third model (M1.3: day) exclusively uses information recorded when the participants were awake, such as HR while awake. All 3 models include an L1 penalty to shrink the coefficient values of features without great explanatory power to 0. Table 4 lists variables that are statistically significant in at least 1 of the 3 models. As outlined in the previous section, logistic regression achieved an accuracy of 65% on a perfectly balanced test set when including features collected while participants were awake and asleep (M1.1).

Across M1.1 to M1.3 in Table 4, observed effects have a constant sign across all models that they are included in, indicating general consistency of calculated effects independent of included input features. In addition to whether a participant woke up during the night, the levels of stress they were exposed to the previous day, the duration of their sleep, and recorded motion while asleep, 8 HR- or HRV-related features significantly affected perceived sleep quality in 1 of the 3 models M1.1-M1.3. In M1.1-M1.3, increased sleep duration and decreased motion while asleep are calculated to affect sleep quality positively—so are increases in HRV while asleep in terms of average SD1, maximum SD2, and maximum SDNN and increases in minimal HR while asleep. In contrast, increases in average SD2 while asleep as well as increases in the ratio of maximum SDNN while asleep to average SDNN while asleep are calculated to affect perceived sleep quality negatively. We found higher levels of stress throughout the previous day and if a participant woke up during the night to affect perceived sleep quality negatively.

Table 4 shows variables that are statistically significant (*P*<.10) in at least 1 logistic regression model for normalized self-reported sleep quality without interaction terms where feature selection was performed for both groups simultaneously but the models were calculated for participants with MS and the control group separately. Positive values increase the chances of better self-reported sleep quality according to the fitted logistic regression model.

**Table 4:** Statistically significant variables for normalized perceived sleep quality for patients with multiple sclerosis and the control group.

	M1.1: night and day as input, model coefficient (*P* value)	M1.2: night as input only, model coefficient (*P* value)	M1.3: day as input only, model coefficient (*P* value)
Mean stress awake	—^a^	—	–0.14 (.04)
HR^b^ minimum ratio awake	0.16 (.03)	—	—
Sleep duration	0.47 (<.001)	0.46 (<.001)	—
Awake at night	–0.58 (<.001)	–0.58 (<.001)	—
Motion asleep	–0.29 (.02)	–0.29 (.02)	—
Minimum HR asleep	—	0.13 (.04)	—
Mean SD1^c^ asleep	0.30 (.004)	0.26 (.01)	—
Minimum SD2^d^ asleep	—	–0.18 (.06)	—
Mean SD2 asleep	–0.47 (.01)	–0.43 (.02)	—
Maximum SD2 asleep	0.08 (.03)	0.06 (.04)	—
Maximum SDNN^e^ asleep	0.51 (<.001)	0.47 (<.001)	—
SDNN maximum ratio asleep	–0.54 (.01)	–0.52 (.01)	—

^a^—: The variable was not included in that respective model (ie, removed during iterative feature selection process).

^b^HR: heart rate.

^c^SD1: SD of distance from the 45° line of the Poincaré plot of consecutive interbeat intervals.

^d^SD2: SD of distance from the –45° line of the Poincaré plot of consecutive interbeat intervals.

^e^SDNN: SD of interbeat intervals.

### Differences Between Patients With MS and the Control Group

**Figure 1:**
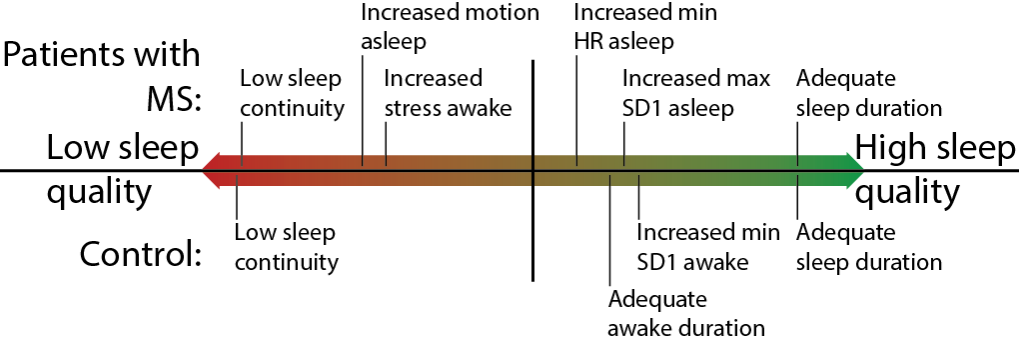
Approximate visualization of the statistically significant effects of M3.1-M3.3 for patients with multiple sclerosis (MS; upper half) and the control group (lower half), as displayed in Table 6. In this visualization, only the order of the effects for each of the 2 groups separately is correct. The distances are not proportional to the calculated effects (Table 6). Sleep continuity refers to “awake at night” in Tables 4-6, that is, whether participants woke up at night normalized across the duration of the study. HR: heart rate; max: maximum; min: minimum; SD1: SD of distance from 45° line of Poincaré plot of consecutive interbeat intervals.

We analyze differences in effects between the control group and participants with MS by computing the 3 logistic regression models M1.1-M1.3 for normalized perceived sleep quality separately for the 2 groups (Table 5). We refer to these models as M2.1-M2.3. They are based on the feature selection performed for M1.1-M1.3, but the statistical significance of effects are calculated for the 2 groups separately, thus analyzing the stability of the calculated effects across the 2 groups. The performance of M2.1 dropped from an accuracy of 65% for M1.1 to 60% accuracy for the control group and 64% for participants with MS. Furthermore, we constructed 3 more logistic regression models, M3.1-M3.3, where the included features are optimized for the control group and the patient group separately (Table 6). Figure 1 visually summarizes Table 6. For the models M2.1-M2.3, the calculated effects for the same variables have the same sign in all cases apart from 2, indicating largely agreeing effects across the subgroups. When optimizing the feature selection for each subgroup, we observe divergence in the included features and an improvement in performance (accuracy increases to 68% for the control group and remains at 64% for participants with MS).

The effect of increases in minimal HR while asleep is calculated to be positive and statistically significant at α=1% in M2.1 and M2.2 for participants with MS but not statistically significant and negative for the control group. The effect of increases in reported stress levels in M2.3 is statistically significant (*P*<.01) for patients with MS and the control group, yet <0.005 in absolute terms for the control group.

In M3.1 and M3.2, sleep duration and being awake at night are selected for both subgroups. For both effects, sign, statistical significance, and general magnitude were the same. We find stress, motion while asleep, minimal HR while asleep, and maximum SD1 while asleep to only significantly affect perceived sleep quality for participants with MS. In contrast, the amount of time spent awake before going to bed and minimal SD1 while awake only affected perceived sleep quality for participants of the control group.

**Table 5:** Differences in statistically significant variables for perceived sleep quality between patients with multiple sclerosis and the control group: simultaneous feature selection.

	2.1: Night and day as input, model coefficient (*P* value)		2.2: Night as input only, model coefficient (*P* value)		M2.3: day as input only, model coefficient (*P* value)	
	Patients with multiple sclerosis	Control group	Patients with multiple sclerosis	Control group	Patients with multiple sclerosis	Control group
Mean stress awake	—^a^	—	—	—	–0.19 (.01)	–0.00 (.001)
HR^b^ minimum ratio awake	0.20 (.01)	0.13 (.39)	—	—	—	—
Sleep duration	0.37 (<.001)	1.21 (.002)	0.40 (<.001)	0.72 (<.001)	—	—
Awake at night	–0.56 (.001)	–1.01 (.06)	–0.57 (.001)	–0.66 (.07)	—	—
Motion asleep	–0.28 (.03)	–0.40 (.21)	–0.29 (.02)	–0.24 (.23)	—	—
Minimum HR asleep	—	—	0.25 (.004)	–0.21 (.19)	—	—
Mean SD1^c^ asleep	0.28 (.01)	0.47 (.10)	0.21 (.08)	0.40 (.004)	—	—
Minimum SD2^d^ asleep	—	—	–0.27 (.02)	–0.09 (.27)	—	—
Mean SD2 asleep	–0.32 (.06)	–1.37 (.09)	–0.47 (.02)	–0.24 (.19)	—	—
Maximum SD2 asleep	0.08 (.04)	0.25 (.21)	−0.31 (.32)	0.08 (.03)	—	—
Maximum SDNN^e^ asleep	0.39 (.001)	1.25 (.07)	0.99 (.06)	0.17 (.007)	—	—
SDNN maximum ratio asleep	–0.47 (.03)	–1.41 (.10)	–0.67 (.01)	–0.17 (.22)	—	—

^a^—: The variable was not included in that respective model (ie, removed during iterative feature selection process).

^b^HR: heart rate.

^c^SD1: SD of distance from the 45° line of the Poincaré plot of consecutive interbeat intervals.

^d^SD2: SD of distance from the –45° line of the Poincaré plot of consecutive interbeat intervals.

^e^SDNN: SD of interbeat intervals.

**Table 6:** Differences in statistically significant variables for perceived sleep quality between patients with multiple sclerosis and the control group: separate feature selection.

	M3.1: night and day, model coefficient (*P* value)			M3.2: night, model coefficient (*P* value)			M3.3: day, model coefficient (*P* value)	
	Patients with multiple sclerosis	Control group	Patients with multiple sclerosis		Control group	Patients with multiple sclerosis		Control group
Mean stress awake	—^a^	—	—		—	–0.19 (.02)		—
Awake duration	—	0.43 (.05)	—		—	—		—
Minimum SD1^b^ awake	—	—	—		—	—		0.48 (.05)
Sleep duration	0.39 (<.001)	0.93 (<.001)	0.42 (<.001)		0.82 (<.001)	—		—
Awake at night	–0.53 (.001)	—	–0.56 (.002)		–0.86 (.06)	—		—
Motion asleep	–0.26 (.02)	—	–0.28 (.02)		—	—		—
Minimum HR^c^ asleep	0.08 (<.001)	—	0.27 (.002)		—	—		—
Maximum SD1 asleep	0.17 (.02)	—	0.18 (.04)		—	—		—

^a^—: The variable was not included in that respective model (ie, removed during iterative feature selection process).

^b^SD1: SD of distance from 45° line of Poincaré plot of consecutive interbeat intervals.

^c^HR: heart rate.

Table 5 shows variables that are statistically significant (*P*<.10) in at least 1 logistic regression model for normalized self-reported sleep quality without interaction terms where feature selection was performed for both groups simultaneously, but the models were calculated for participants with MS and the control group separately. Positive values increase the chances of better self-reported sleep quality according to the fitted logistic regression model.

Table 6 shows variables that are statistically significant (*P*<.10) in at least 1 logistic regression model for normalized self-reported sleep quality without interaction terms where feature selection was performed for participants with MS and the control group separately. Positive values increase the chances of better self-reported sleep quality according to the fitted logistic regression model.

### Correlating Input Factors of the Logistic Regression Models

Multiple input factors of the different models for normalized perceived sleep quality (M3.1-M.3.3) correlated statistically significantly (Multimedia Appendix 3). To highlight shared information content between multiple models, Multimedia Appendix 4 displays Pearson correlations for features selected for M3.1-M3.3, where the feature selection and computation of the statistical significance of effects were performed separately for participants with MS and the control group. The correlations are calculated separately for participants with MS and the control group, allowing for comparison of the relation between the 2 groups. In total, 2 pairs of input variables for M3.1-M3.3 correlated particularly strongly with a correlation coefficient of –0.41 to –0.49 (*P*<.001): the pair of duration of sleep and the duration participants spent awake before going to bed and the pair of average SD1 while asleep and minimal HR while asleep. Interestingly, there are also 3 pairs where the difference in correlation between the control group and the participants with MS was particularly high. First, minimal HR while asleep and motion while asleep correlated with a coefficient of .11 for participants with MS but with a coefficient of –0.09 for the control group. Second, minimal HR while asleep and recorded stress levels correlated with a correlation coefficient of –0.17 for participants with MS but with a coefficient 0.06 for the control group, indicating a difference in response to stress. Third, the duration participants spent awake before going to bed and the recorded levels of stress correlated with a coefficient of –0.12 for participants with MS but 0.09 for the control group, further indicating a potential difference in behavior as a reaction to stress.

## Discussion

### Principal Findings

In this study, we analyzed how the ANS, the cardiovascular system, stress, activity, and demographic information affect perceived sleep quality for patients with MS and a control group. Model performances suggest that relative changes in perceived sleep quality per participant can indeed predict perceived sleep quality using a combination of HRV metrics, activity data, and stress (M1.1-M3.3). Generally, we find greater HRV to significantly improve perceived sleep quality. However, we find that activation of stress response (high sympathetic and low parasympathetic activity), similar to higher levels of perceived stress, significantly decreases perceived sleep quality. For the control group, this effect is less severe.

With stress levels and sleep duration, we find predictors particularly important for the sleep quality of patients with MS that can be at least partially acted upon to improve perceived sleep quality. However, calculated effects regarding signals that are not directly controllable (eg, HRV) are much more difficult to translate into actionable recommendations. For the effects of HR and HRV, further studies are needed to better understand the underlying drivers of these signals and how they can be acted upon.

### Effects of HRV on Sleep Quality

We found various HRV metrics to be suitable predictors for perceived sleep quality. In particular, increased SD1 metrics positively impacted normalized perceived sleep quality across M1.1-M3.3, highlighting their consistency for patients with MS as well as the control group. However, the calculated effects of SD2 and SDNN seemed contradictory and inconsistent in M1.1-M1.3 and were not selected when the automated feature selection procedure was conducted separately for participants with MS and the control group for M3.1-M3.3.

There are various factors that influence ANS activity and thus also HRV metrics. While participants are awake, physical activity, stress, overall mood, and deep breathing [53,54] might impact HRV metrics. In the long run, ANS activity is also affected by MS disease progression [16].

While asleep, a possible explanation for the calculated effects of increased activity of the sympathetic and parasympathetic nervous system is their behavior during rapid eye movement (REM) and non-REM sleep phases and their connection to stress. HRV fluctuates strongly between different phases of sleep [25,55-57] and is particularly high during REM sleep. More time spent in REM sleep phases was found to increase subjective sleep quality and also cognitive performance [58], which matches the calculated effects regarding the sympathetic and parasympathetic nervous system. While the activity of the former increases during REM sleep compared to non-REM sleep, the activity of the latter decreases during REM sleep phases [59]. Furthermore, the sympathetic nervous system regulates the fight-or-flight response and gives an indication of stress levels. The negative effect of increases in sympathetic activity and positive effect of increased parasympathetic activity on sleep quality might thus indicate that participants experienced stress throughout the day, which carried on into their sleep (activated stress response), or that participants went through stressful experiences during their REM sleep, which might again be impacted by experienced stress while awake. As outlined in M1.3-M3.3, we found increased stress to reduce perceived sleep quality, thus matching the effects outlined above.

### Effects of MS Diseases Status on Sleep Quality

Symptoms of severe MS significantly decreased perceived sleep quality. However, we did not find significant differences in subjective sleep quality between participants diagnosed with MS and the control group. This indicates that MS itself does not affect perceived sleep quality. However, scoring high on the ARMSS, MSSS, or EDSS scale and affection of the spinal cord or ANS resulted in significantly worse sleep for patients with MS. Thus, symptoms that were found to decrease general quality of life for patients with MS also contribute to lower perceived sleep quality, matching previous studies [3,16,30,60].

### Effects of Sleep Duration and Awake Duration on Sleep Quality

We generally found increased sleep duration to positively affect perceived sleep quality. The effects were statistically significant for participants with MS as well as the control group across M1.1-M3.3 and are rather unsurprising. This is a further indication of the importance of an adequate sleep schedule to achieve high-quality sleep and increase quality of life. For patients with MS as well as healthy individuals, this offers an opportunity to improve their sleep quality and subsequently quality of life. Furthermore, at least for the control group, longer times spent awake before going to bed positively affected their perceived quality of sleep. This seems in contrast to the positive effect of longer sleep duration; however, this might indicate that too little time spent awake negatively impacts perceived sleep quality. The 2 effects of longer sleep duration and longer awake duration thus highlight the need of a balance between the time spent awake and asleep.

### Effects of Stress on Sleep Quality

We found stress levels (self-reported) to have a statistically significant negative impact on perceived sleep quality. For participants with MS, stress impacted perceived sleep quality more strongly than it did for the control group (Table 6). Furthermore, for participants with MS, stress correlated significantly negatively with awake duration and minimal HR while asleep. This indicates that stress more severely affects patients with MS, which is even measurable using their minimal HR while asleep. The significant negative correlation of stress to awake duration for participants with MS, which is positive but statistically insignificant for the control group, might indicate that stress even impacted the daily routine of participants with MS.

These effects are consistent with existing literature [26-28,61]. For patients with MS, several studies suggest that increased stress increases the chances of relapse [62] and influences inflammatory activity [63]. Furthermore, the ANS is a stress response system. The more severe effect of stress on sleep quality for participants with MS might thus be another symptom of a dysfunctional ANS.

While we do not find reported stress levels to impact the sleep quality for the control group, we observe negative effects of increases in sympathetic activity (SDNN and SD2) in M2.1-M2.3 for patients with MS as well as the control group. The sympathetic nervous system controls the fight-or-flight response, and stress might cause an activated stress response, which is characterized by increased sympathetic activity and decreased parasympathetic activity. This indicates a negative effect of stress on perceived sleep quality for both groups, although not observable through reported stress levels for the control group.

While we calculate similar effects for perceived stress and objective measures of stress (ie, an activated stress response), perceived stress ratings and objective assessments of stress do not have to align [64]. Comparisons between objective and subjective assessments of stress must thus be treated carefully.

### Effects of Motion (Steps) on Sleep Quality

An increase in recorded arm motion between initially falling asleep and waking up in the morning significantly decreased perceived sleep quality. The recorded motion records both steps of participants (eg, to use the bathroom) and general movement while asleep due to low sleep continuity or a sleep disorder, such as period leg movement disorder. Period leg movement disorder affects 8% to 11% of the population [65] and around 25% of patients with MS [13,18,66]. Generally, excessive motion indicates disruption of sleep. Recorded motion during the night furthermore correlates significantly with being awake at night (*r*=0.22; *P*=.004). The negatively calculated effects for increases in both variables match existing literature about sleep continuity [67] as well as compartment 5 of the Pittsburgh Sleep Quality Index [24].

### Effects of HR on Sleep Quality

Despite various HR-based variables being included in M1.1-M2.3, only minimal HR while asleep significantly affected perceived sleep quality for patients with MS in M3.1-M3.2. While the effect of increases in minimal HR seems consistent for patients with MS, it seems to have no effect on sleep quality for the control group. This highlights a difference in the effect of cardiovascular activity between the 2 groups.

### Effects of Weather (Temperatures) on Sleep Quality

We did not find temperature to affect perceived sleep quality in our study, contradicting previous research about the influence of weather on sleep quality [68] and temperatures on the well-being of patients with MS [69]. However, past research suggests that mainly the room temperature when falling asleep impacts sleep [70]. The overall temperature outside, as recorded during our study, is only a (poor) estimate of room temperature.

### Relation Between Demographic Information and Sleep Quality

Female participants slept significantly better (subjectively) compared to male participants in our study. This is contradicting existing literature on objective sleep quality where female participants were found to sleep significantly worse and also shorter than male participants [71,72]. Furthermore, women were also found to be 1.41 times more likely to experience insomnia compared to men [73].

Matching previous studies [71,74], we found age to correlate strongly with motion while asleep and being awake at night (Pearson correlation of *r*=0.31 and *r*=0.23, respectively, with *P*<.001). Both factors are calculated to significantly reduce sleep quality (M1.1-M3.2), which matches existing literature about reduced sleep continuity of older individuals [74].

### Limitations

Our study has several limitations that question the generalizability and immediate clinical applicability of our results.

First, because we collect ANS activity passively, we cannot control all the factors that influence ANS activity and might confound our results. In the long run, ANS activity is influenced by disease progression for patients with MS [16]. Temporarily, ANS activity might be influenced by deep breathing exercises, shock, mood, physical exercise, and generally any type of stressor [53,54]. As we aggregate ANS activity over multiple hours when participants are either awake or asleep, it seems unlikely that we capture either very short bursts of ANS activity or long-term trends caused for instance by MS disease progression. The multitude of factors that influence ANS activity, however, only allow for hypothesis about the exact causes of the effects we observe. This uncertainty makes the translation into actionable clinical recommendations difficult.

Second, the translation of our findings into clinical recommendations is further limited because many effects found to be important for sleep quality estimation are based on signals collected while participants are asleep. Our analysis does not reveal what actions cause variables such as minimal HR while asleep to differ. Thus, we can only provide recommendations for variables such as stress or sleep duration.

Third, despite the diversity within our study population, it is unlikely that it covers the diverse range of MS disease traits. As we recruited patients with MS solely at the neuroimmunology department of the University Hospital Zurich, our study is effectively limited to Switzerland. While our findings regarding ANS activity might generally be assumed to generalize to patients with MS outside of Switzerland, there are likely several confounders that bias our findings due to specifics of the life in Switzerland, its health care sector, or the genetic traits of Switzerland’s population. As part of a larger and more representative study, it would also be possible to stratify for disease progression and ANS dysfunction to investigate the robustness of our findings toward particularly severe cases of MS with a highly dysfunctional ANS.

Finally, we investigate self-reported sleep quality, which does not have to align well with objective measures of sleep quality [75]. While we find objective measures of sleep quality to be strong predictors of perceived sleep quality and also normalize perceived sleep quality ratings per participant to remove intrasubject variability, our results have to be treated with care. Similarly, we investigate perceived stress ratings, which do not form a passive and objective measure of stress. Again, we find similar effects of objective measures of stress (ie, an activated stress response at night) and self-reported stress levels and also normalized stress ratings per participant. However, self-reported stress levels do not have to be consistent, and a comparison between self-reported and objectively assessed stress levels is difficult [64].

Generally, all the points above outline that a larger study is needed to confirm our findings and hopefully derive actionable insides. This will hopefully allow to derive what actions cause the observed changes in signals that are outside participants’ direct control. We hope our study lays the basis for such larger efforts.

### Future Research

In addition to addressing what is outlined in the Limitations section, there are multiple avenues worth exploring for future research.

First, we believe a better understanding of the ANS of each patient with MS would prove most valuable. This might be achieved via imaging, particularly through connectomes that provide a mapping of the nervous system’s connectivity. Given recent successes in connectome-based predictive modeling [76], the mapping of connectomes of patients with MS to perceived sleep quality might prove an interesting first step. Similarly, an analysis of the location of lesions in the CNS might help to explain why the ANS of patients with MS might relate differently to perceived sleep quality. Subsequently, this might help to identify different subgroups of patients with MS, who might have to be treated differently to improve their sleep quality and quality of life.

Second, we believe incorporating information about sleep stages into the analysis might prove most valuable. As outlined in the Effects of HRV on Sleep Quality section, ANS activity fluctuates between different sleep stages. Therefore, through or simple aggregation across the whole night, important patterns might currently be neglected.

Combined with large representative studies that are also able to establish causal relationships between participants’ behavior and changes in bio signals, we believe this would paint a precise and representative picture of the connection between ANS activity of sleep quality more generally for patients with MS and heathy individuals alike.

Third, we believe a longitudinal study that captures potential disease progression in patients with MS would provide valuable insights into how sleep quality and ANS activity might change based on different stages of MS, including relapses. It would be most interesting to include interventions in such a study design to verify the causality of our results, for example, intervening on participants sleep duration.

Fourth, low sleep quality is a symptom of various neurological conditions such as Parkinson, epilepsy, or Huntington. Some of the results we derived might translate and prove valuable also to patients with other neurological conditions.

### Conclusions

The results we present are 3-fold. First, we have found new predictors for the perceived sleep quality of patients with MS as well as healthy individuals, which are conveniently measurable using wearable sensors. We thereby gained a better understanding of the impact of HRV on sleep quality and the differences in effect for patients with MS, namely, an activated stress response (lower parasympathetic activity and higher sympathetic activity) while asleep impacts perceived sleep quality negatively. However, the activity of the parasympathetic nervous system has greater impact on perceived sleep quality than sympathetic activity, especially for healthy individuals.

Second, we found the disease state of patients with MS to impact perceived sleep quality. In particular, patients with MS whose ANS was dysfunctional; whose spinal cord was affected; or who scored highly on the MSSS, ARMSS, or EDSS reported significantly lower sleep quality than patients with MS whose ANS was not dysfunctional; whose spinal cord was unaffected; and who scored lower on the MSSS, ARMSS, or EDSS, respectively.

Third, for binary classification problems using medical sensor data, we provide further evidence for the use of more conventional models that are interpretable as well as explainable over state-of-the-art black box models. While GAMs outperformed all other models, GLMs performed similar to boosted tree ensemble classifiers or support vector machines and outperformed neural networks.

## Appendix

Multimedia Appendix 1 Patients with multiple sclerosis on disease-modifying therapy.

Multimedia Appendix 2 Patients with multiple sclerosis on medication that is known to affect heart rate variability.

Multimedia Appendix 3 Distribution of sleep quality ratings.

Multimedia Appendix 4 Correlation between input variables to models M3.1-M3.3.

## Abbreviations

ANS: autonomic nervous system
AUC: area under the curve
DMT: disease-modifying treatment
EDSS: Expanded Disability Status Scale
GAM: generalized additive model
GLM: generalized linear model
HR: heart rate
HRV: heart rate variability
IBI: interbeat interval
MS: multiple sclerosis
REM: rapid eye movement
SDNN: SD of interbeat intervals

## Data Availability

The data sets generated during and analyzed during this study are available from the corresponding author on reasonable request. Accompanying code is available through the GitHub repository [77].
