# Supplementary material for: Assessing the Role of the Autonomic Nervous System as a Driver of Sleep Quality in Patients With Multiple Sclerosis: Observation Study
Source: JMIR Neurotechnol. 2024 Aug 21;3:e48148. doi: 10.2196/48148 (PMC12671311; doi:10.2196/48148)
Supplement: Multimedia Appendix 1 [file neuro_v3i1e48148_app1.docx]

## Multimedia Appendix 1: MS patients on disease-modifying therapy (DMT)

| medication | MS patients |
| --- | --- |
| None | 10 |
| Dimethylfumarat | 5 |
| Natalizumab | 11 |
| Ocrelizumab | 11 |
| Ozanimod | 2 |
| Rituximab | 3 |
| Siponimod | 1 |
| Teriflunomid | 1 |
| aHSCT | 9 |

Number of MS patients on disease-modifying therapy (DMT) per medication.
