# Supplementary material for: Assessing the Role of the Autonomic Nervous System as a Driver of Sleep Quality in Patients With Multiple Sclerosis: Observation Study
Source: JMIR Neurotechnol. 2024 Aug 21;3:e48148. doi: 10.2196/48148 (PMC12671311; doi:10.2196/48148)
Supplement: Multimedia Appendix 2 [file neuro_v3i1e48148_app2.docx]

## Multimedie Appendix 2: MS patients on HRV-altering medication

| medication | MS patients |
| --- | --- |
| None | 36 |
| Betmiga | 3 |
| Citalopram | 1 |
| Cymbalta | 3 |
| Duloxetin | 1 |
| MetoZerok | 1 |
| Minoxidil | 2 |
| Ozanimod | 2 |
| Ritalin | 1 |
| Siponimod | 1 |
| Tamsulosin | 1 |
| Trittico | 1 |

Number of MS patients that take medication known to affect HRV metrics per medication.
