# Supplementary figures and images for: Assessing the Role of the Autonomic Nervous System as a Driver of Sleep Quality in Patients With Multiple Sclerosis: Observation Study
Source: JMIR Neurotechnol. 2024 Aug 21;3:e48148. doi: 10.2196/48148 (PMC12671311; doi:10.2196/48148)

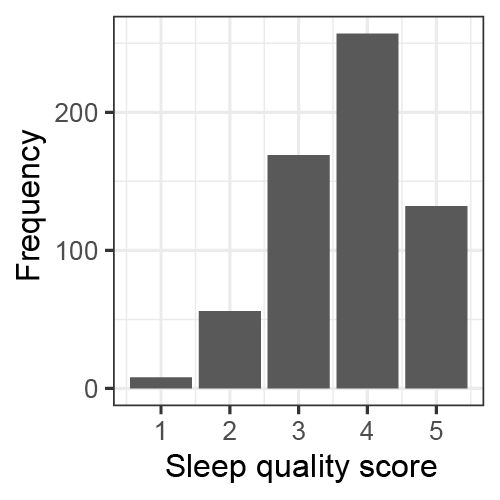

Supplement: Multimedia Appendix 3 [file neuro_v3i1e48148_app3.png]
