# Supplementary material for: Assessing the Role of the Autonomic Nervous System as a Driver of Sleep Quality in Patients With Multiple Sclerosis: Observation Study
Source: JMIR Neurotechnol. 2024 Aug 21;3:e48148. doi: 10.2196/48148 (PMC12671311; doi:10.2196/48148)
Supplement: Multimedia Appendix 4 [file neuro_v3i1e48148_app4.docx]

## Multimedia Appendix 1: Table A.1

| awake duration | Co | **0.09** | *−*0*.*12 | *−*0*.*46 | *−*0*.*05 | 0.12 | *−*0*.*04 | *−*0*.*08 |
| --- | --- | --- | --- | --- | --- | --- | --- | --- |
|  | MS | *−***0***.***12** | *−*0*.*07 | *−*0*.*41 | *−*0*.*18 | 0.26 | *−*0*.*05 | *−*0*.*12 |
|  |  |  |  |  |  |  |  |  |
| min SD1 awake | Co | *−*0*.*02 | 0.03 | 0.03 | 0.02 | *−*0*.*11 | 0.17 |  |
|  | MS | 0.1 | *−*0*.*08 | 0.05 | *−*0*.*08 | *−*0*.*21 | 0.27 |  |
|  |  |  |  |  |  |  |  |  |
| mean SD1 asleep | Co | *−*0*.*02 | 0 | 0.1 | 0.05 | *−*0*.*49 |  |  |
|  | MS | *−*0*.*07 | *−*0*.*19 | *−*0*.*02 | *−*0*.*16 | *−*0*.*42 |  |  |
|  |  |  |  |  |  |  |  |  |
| min HR asleep | Co | **0.06** | *−***0***.***09** | *−*0*.*18 | *−*0*.*01 |  |  |  |
|  | MS | *−***0***.***17** | **0.11** | *−*0*.*10 | *−*0*.*03 |  |  |  |
|  |  |  |  |  |  |  |  |  |
| awake duration | Co | 0.02 | 0.22 | 0.06 |  |  |  |  |
|  | MS | 0.02 | 0.26 | 0.04 |  |  |  |  |
|  |  |  |  |  |  |  |  |  |
| asleep duration | Co | *−*0*.*05 | 0.27 |  |  |  |  |  |
|  | MS | 0.06 | 0.25 |  |  |  |  |  |
|  |  |  |  |  |  |  |  |  |
| motion asleep | Co | *−*0*.*08 |  |  |  |  |  |  |
|  | MS | *−*0*.*09 |  |  |  |  |  |  |
|  |  | stress awake | motion asleep | sleep duration | awake at night | min HR asleep | mean SD1 asleep | min SD1 awake |

Underlined correlation coefficients are statistically significant at a level of α = 5%. Bold values indicate that the sign of the correlation coefficient differs between the two groups and the difference is greater than 0.2, or one correlation coefficient is twice as large as the other one with a difference of at least 0.2.
